# Integrated Metabolomic and Transcriptomic Analysis Reveals the Underlying Antibacterial Mechanisms of the Phytonutrient Quercetin-Induced Fatty Acids Alteration in *Staphylococcus aureus* ATCC 27217

**DOI:** 10.3390/molecules29102266

**Published:** 2024-05-11

**Authors:** Haihua Yuan, Hang Xun, Jie Wang, Jin Wang, Xi Yao, Feng Tang

**Affiliations:** Key Laboratory of National Forestry and Grassland Administration/Beijing for Bamboo & Rattan Science and Technology, International Centre for Bamboo and Rattan, Beijing 100102, China; yuanhh1022@163.com (H.Y.); xunhang@icbr.ac.cn (H.X.); wangjiekobe@163.com (J.W.); wangjin@icbr.ac.cn (J.W.); yaoxi@icbr.ac.cn (X.Y.)

**Keywords:** quercetin, *Staphylococcus aureus*, antibacterial mechanisms, fatty acid biosynthesis, β-ketoacyl-ACP reductase, metabolomics, transcriptomics

## Abstract

The utilization of natural products in food preservation represents a promising strategy for the dual benefits of controlling foodborne pathogens and enhancing the nutritional properties of foods. Among the phytonutrients, flavonoids have been shown to exert antibacterial effects by disrupting bacterial cell membrane functionality; however, the underlying molecular mechanisms remain elusive. In this study, we investigated the effect of quercetin on the cell membrane permeability of *Staphylococcus aureus* ATCC 27217. A combined metabolomic and transcriptomic approach was adopted to examine the regulatory mechanism of quercetin with respect to the fatty acid composition and associated genes. Kinetic analysis and molecular docking simulations were conducted to assess quercetin’s inhibition of β-ketoacyl-acyl carrier protein reductase (FabG), a potential target in the bacterial fatty acid biosynthesis pathway. Metabolomic and transcriptomic results showed that quercetin increased the ratio of unsaturated to saturated fatty acids and the levels of membrane phospholipids. The bacteria reacted to quercetin-induced stress by attempting to enhance fatty acid biosynthesis; however, quercetin directly inhibited FabG activity, thereby disrupting bacterial fatty acid biosynthesis. These findings provide new insights into the mechanism of quercetin’s effects on bacterial cell membranes and suggest potential applications for quercetin in bacterial inhibition.

## 1. Introduction

Microbiological contamination can significantly affect the nutritional value of food. The food industry’s reliance on synthetic preservatives for extending shelf life is facing growing health and safety concerns [1]. Scientists worldwide are exploring safe, effective alternatives, like plant-derived products. Over billions of years, plants have developed a vast array of natural products essential for their growth, development, and defense [2]. Among these, flavonoids have exhibited significant antibacterial properties against foodborne pathogens, like *Escherichia coli*, *Salmonella enteritidis*, and *Staphylococcus aureus*, making them promising agents for ensuring food safety [3,4]. Furthermore, flavonoids, due to their diverse bioactivities and potential to promote health and reduce the incidence of chronic diseases, such as coronary heart disease, diabetes and cancer, are a major area of research in the field of food and nutrition science [5,6]. Therefore, the use of phytonutrients, such as flavonoids, in food preservation offers unique antimicrobial properties, low toxicity, and the ability to preserve food flavor and improve food nutrition [7,8].

The evaluation of dihydromyricetin, naringenin, kaempferol, and many other flavonoids as potential food preservatives has been recently reviewed. These flavonoids are recognized for their ability to exert antimicrobial effects by altering the fatty acid composition of foodborne pathogens’ cell membranes [9,10]. Fatty acids, being key components of membrane phospholipids, directly influence the physical properties of the cell membrane. Bacteria can adapt their membrane compositions in terms of diversity of the fatty acid chain length and saturation levels in response to environmental changes. This is a key strategy for survival and proliferation under different conditions [11]. In addition to their fundamental role in the structure of cell membranes, fatty acids are essential biomolecules for bacterial energy, signaling, and the expression of virulence factors [12]. The interference of flavonoids with bacterial fatty acids suggests highly attractive antibacterial targets and mechanisms of action. The canonical and conserved fatty acid biosynthesis (FAS-II) pathway is the sole endogenous means for bacterial fatty acid biosynthesis and involves the catalysis of fatty acid synthase for long-chain fatty acid production [13]. The type-II fatty acid synthase employed by prokaryotes and plants operates through a multi-enzyme system formed by non-covalent bonds. Each enzyme contributes to a diverse array of reactions, which vary from the type-I fatty acid synthase in animals and fungi. The distinctiveness of the FAS-II pathway offers a strategy for developing antibacterial agents that specifically affect bacteria without harming eukaryotic cells, such as human cells [14].

The inhibition of mutant forms of several critical enzymes in the FAS-II pathway results in bacterial death, validating these enzymes’ potential for use as targets for antibacterial agent development [15]. GlaxoSmithKline conducted the largest documented target-based antibiotic high-throughput screening of activity; of the five most valuable leads identified, two targeted enzymes were related to the bacterial fatty acid biosynthesis system [16]. A highlight in the field of FAS-II pathway research is the discovery of many potential natural product inhibitors. Cerulenin [17], platencin [18], platensimycin [18], and thiolactomycin [19] are examples of natural products that specifically inhibit β-ketoacyl-ACP synthase I/II (FabF/B), β-ketoacyl-ACP synthase III (FabH), and β-enoyl-ACP reductase (FabI). These compounds have demonstrated antibacterial efficacy against a wide range of Gram-negative and Gram-positive bacteria [20]. β-Ketoacyl-ACP reductase (FabG) is the only enzyme without isozymes in the fatty acid biosynthesis pathway of many pathogenic bacteria, such as *E. coli* and *S. aureus*, and it is highly conserved. Although it is not the rate-limiting enzyme in this pathway, it is still an important candidate target [21,22]. Onions, grapes, maple leaves, and galangal extracts, as well as monomer plant phenolics, such as (-)-epigallocatechin gallate (EGCG), (-)-catechin gallate (CG), chlorogenic acid, and a series of flavonoids, have been found to exhibit inhibitory activity against FabG [23], suggesting possible antibacterial mechanisms for these dietary nutraceuticals and their potential use as food preservatives.

Although it has been demonstrated that flavonoids can affect the bacterial fatty acid composition, their specific targets and modes of regulation of the bacterial fatty acid biosynthesis pathway remain unclear. This is because studies have mainly been carried out at the cellular level or by identifying key metabolites, genes, or enzymes in specific pathways on an individual basis. The reaction of bacteria to the stress induced by an antibacterial agent is not a series of simple or isolated responses but a complex biological process involving the dynamics of multiple pathways and regulatory networks. These pathways interact with each other to form a highly coordinated and interdependent system that maintains bacterial survival and adaptation [24]. Merely measuring the content of certain metabolites, gene expression, or enzyme activity is insufficient.

Quercetin (for the molecular structure, see Figure S1) is a widely distributed flavonol in nature, and its nutritional properties have been extensively studied [25,26]. Meanwhile, its effect on the bacterial cell membrane has been documented [27,28]. Quercetin serves as a valuable model for studying flavonoids’ effects on bacterial fatty acids due to its safety and availability. In this study, we employed a systems biology approach to evaluate the inhibitory effects of quercetin on *S. aureus*. An integrated metabolomic and transcriptomic analysis was used to identify the regulatory mechanism of quercetin’s influence on the bacterial fatty acid composition. The mode of action of quercetin inhibition with respect to the target proteins was further elucidated through an in vitro enzyme activity assay, a molecular docking simulation, and the study of inhibition kinetics. These findings would provide a scientific basis for the development of the phytonutrient quercetin as a new strategy for controlling foodborne pathogens.

## 2. Results

### 2.1. Effect of Quercetin on S. aureus Cells

#### 2.1.1. Morphological and Cell Membrane Integrity Changes

**Figure 1:**
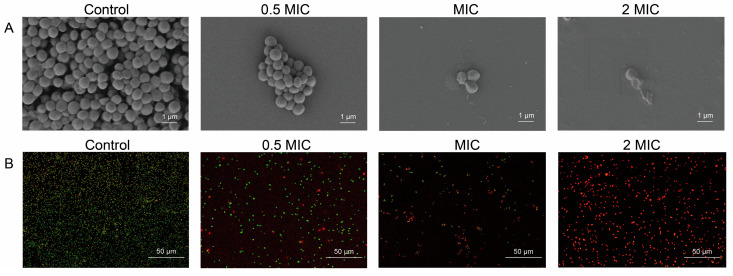
Quercetin-induced cell damage in *Staphylococcus aureus* ATCC 27217. (**A**) Scanning electron microscopy images of *S. aureus* treated with quercetin. (**B**) Confocal laser scanning microscopy images of *S. aureus* treated with quercetin; after fluorescent staining by acridine orange and propidium iodide, live bacterial cells are shown with green fluorescence, and dead bacterial cells with compromised membranes are shown with red fluorescence. MIC, the minimum inhibitory concentration of quercetin against *S. aureus* ATCC 27217, which was found to be 0.5 mg/mL.

The minimum inhibitory concentration (MIC) of quercetin against *S. aureus* ATCC 27217 was found to be 0.5 mg/mL. The morphological changes in *S. aureus* cells treated with quercetin were studied using scanning electron microscopy (SEM). Figure 1A shows that the cells in the control group, which was not subjected to quercetin treatment, exhibited a spherical shape with a smooth surface. However, in the presence of 0.5 MIC quercetin, the treated bacterial cells appeared to disperse, presenting rougher surfaces. Membrane atrophy and corrugation were observed in the bacterial cells treated with quercetin at the MIC. After treatment with 2 MIC of quercetin, *S. aureus* cells exhibited significant membrane deformation, rupture, and blebbing.

To investigate the permeability and integrity of the cell membrane of *S. aureus* after quercetin treatments, confocal laser scanning microscopy (CLSM) using the fluorescent dyes acridine orange (AO) and propidium iodide (PI) was conducted. AO is a nucleic acid stain that is able to penetrate bacterial cells, bind to the DNA, and produce green fluorescence. PI, on the other hand, selectively stains dead bacterial cells with compromised membranes and produces red fluorescence [29]. It can be seen from Figure 1B that the cells in the control group exhibited almost entirely green fluorescence and almost no red fluorescence, suggesting that the membranes of the cells were intact. After treatment with quercetin at 0.5 MIC, a small amount of red fluorescence was observed, indicating that some cell membranes were damaged and their permeability was altered by quercetin. As the concentration of quercetin increased to MIC, the red fluorescence became brighter, while the green fluorescence became darker. When the cells were treated with 2 MIC of quercetin, a significant increase in the red fluorescence intensity was observed. This implied that the cell membrane was more damaged.

#### 2.1.2. Protein and Nucleic Acid Leakage

**Figure 2:**
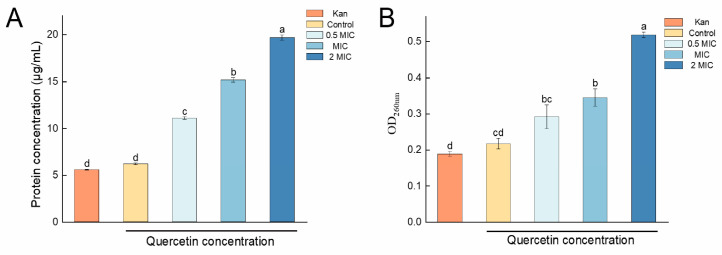
Effects of quercetin treatment on the (**A**) protein and (**B**) nucleic acid leakage of *S. aureus*. Kan, 0.25 mg/L kanamycin sulfate-treated group. OD_260nm_, the optical density at 260 nm. Error bars denote the standard deviations (SD) from three repeated experiments. Different letters above the columns indicate significant differences (*p* < 0.05).

Cell membrane damage usually leads to the release of intracellular macromolecules, such as proteins and nucleic acids [30]. In this study, the extent of cell membrane damage to *S. aureus* treated with quercetin was assessed by measuring the leakage of proteins and nucleic acids. The treatment with quercetin at 0.5 MIC caused protein leakage to increase significantly, rising from 6.19 μg/mL to 11.07 μg/mL (*p* < 0.05), compared to the blank control (Figure 2A). When the concentration of quercetin was increased to 1 and 2 MIC, the protein leakage from *S. aureus* further increased to 15.16 μg/mL and 19.66 μg/mL, respectively. The nucleic acid concentration was expressed as the optical density at 260 nm (OD_260nm_) value. As shown in Figure 2B, in the control groups, very little nucleic acid leakage was detected, indicating that the cell membranes were in a normal condition. However, treatment with quercetin at concentrations of 0.5, 1, and 2 MIC resulted in 1.35-, 1.59-, and 2.39-fold increases in the rates of nucleic acid leakage from *S. aureus* compared to the blank control, demonstrating severe damage to the cell membrane.

#### 2.1.3. Intracellular ATP and Pyruvate Levels

**Figure 3:**
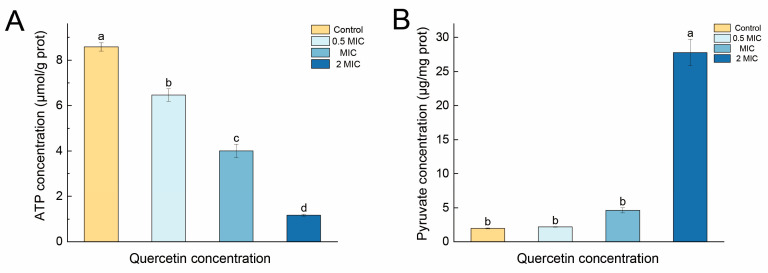
Effects of quercetin treatment on the intracellular (**A**) ATP and (**B**) pyruvate levels of *S. aureus*. prot, protein content. Error bars denote the standard deviations (SD) from three repeated experiments. Different letters above the columns indicate significant differences (*p* < 0.05).

Another method for evaluating cell damage is to determine the intracellular level of adenosine 5′-triphosphate (ATP) [31]. ATP is essential for the growth and reproduction of bacteria. In this study, the ATP concentration was determined via phosphomolybdic acid colorimetry. A concentration-dependent effect was observed in the *S. aureus* cells treated with quercetin (Figure 3A). In the cells treated with 0.5 MIC of quercetin, there was a statistically significant decrease in the ATP content to 6.46 µmol/g protein content (prot), with higher concentrations (1 and 2 MIC) resulting in more pronounced drops in the ATP content to 4.01 µmol/g prot and 1.17 µmol/g prot, respectively.

Pyruvate is the end product of glycolysis and plays a crucial role in cellular energy metabolism [32]. Figure 3B shows that quercetin treatment affected the intracellular pyruvate concentration in *S. aureus*. The pyruvate concentration in the blank control group was 1.97 µg/mg prot. After treatment with quercetin at 0.5 and 1 MIC, the pyruvate concentrations increased to 2.19 µg/mg prot and 4.63 µg/mg prot, respectively. Remarkably, the treatment with 2 MIC of quercetin led to a significant increase in the pyruvate level in *S. aureus*, reaching 27.79 µg/mg prot. Combined with the marked decrease in intracellular ATP, the results indicate a severe abnormality in energy metabolism in *S. aureus*.

The results of the investigation into the effects of quercetin on *S. aureus’* protein and nucleic acid leakage, as well as intracellular ATP and pyruvate levels, were consistent with the SEM and CLSM observations, confirming that quercetin disrupts *S. aureus’* cell membrane integrity, increases cell membrane permeability, and thereby inhibits the growth of *S. aureus* by affecting its membrane function.

### 2.2. Metabolite Accumulation Analysis of S. aureus in Response to Quercetin Treatments

#### 2.2.1. Identification and Quantification of Fatty Acids

As the function of the bacterial cell membrane heavily relies on its fatty acid composition, we first identified the fatty acids in the *S. aureus* cells treated with quercetin using gas chromatography–mass spectrometry (GC–MS), in comparison to reference standards. A total of 14 fatty acids were detected (Table 1), of which 12 were saturated (SFA) and two were unsaturated (UFA). Except for myristic acid and pentadecanoic acid, the contents of the remaining 12 detected fatty acids were up-regulated under the MIC quercetin treatment and down-regulated under the 2 MIC treatment. Accordingly, compared to the control group, the total fatty acid content of the MIC-treated group slightly increased from 130.61 µg/mg prot to 149.42 µg/mg prot, while the 2 MIC treatment resulted in a significant decrease to 85.61 µg/mg prot. From the perspective of chain saturation, palmitic acid and stearic acid, both saturated fatty acids, along with oleic acid, an unsaturated fatty acid, had the highest content, and their variation levels differed. After treatment with the MIC of quercetin, the content of palmitic acid and stearic acid increased by 17.08% and 20.78%, respectively, while that of oleic acid increased by 12.96%. The total ratio of unsaturated fatty acids to saturated fatty acids (1.13) did not significantly change compared to the control group (1.16). However, with the 2 MIC quercetin treatment, the content of palmitic acid and stearic acid decreased by approximately 50%, while that of oleic acid decreased by only 22.33%, resulting in a 50.08% increase in the total UFA/SFA ratio to 1.75. Typically, cell membranes with a higher UFA/SFA ratio exhibit higher fluidity [33].

#### 2.2.2. Metabolomic Profiling

**Figure 4:**
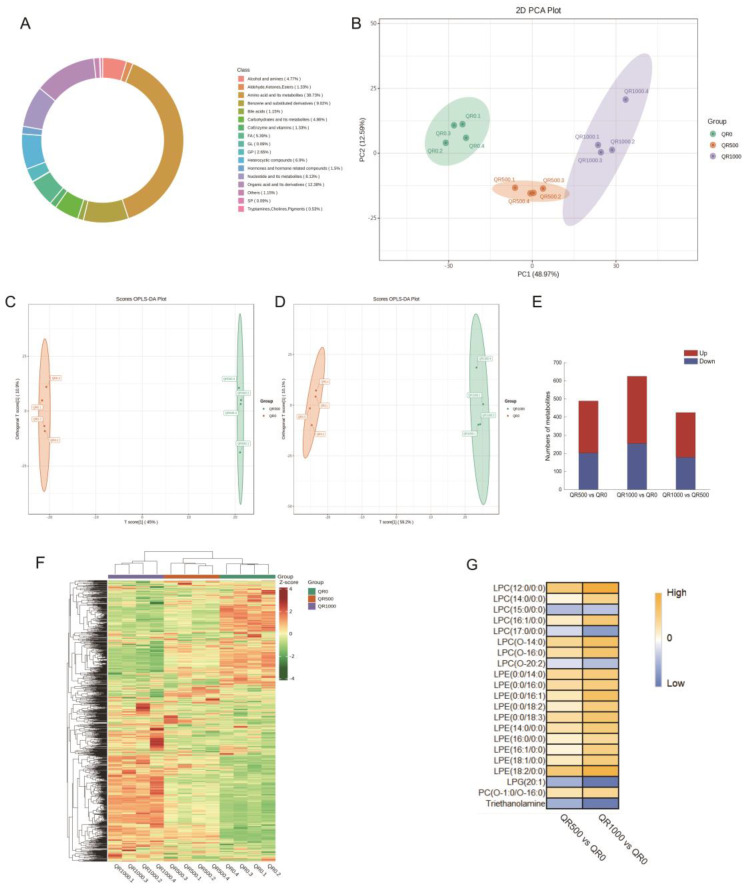
Metabolomic profiling and differential accumulated metabolite (DAM) analysis of *S. aureus* ATCC 27217 under different quercetin treatments. (**A**) Classification of metabolites. (**B**) Principle component analysis (PCA) score plot. (**C**,**D**) Orthogonal partial least squares discriminant analysis (OPLS-DA) score plots. (**E**) The number of up- and down-regulated DAMs in different comparisons. (**F**) Clustering heatmap of the DAMs. (**G**) Heatmap of metabolites related to the glycerophospholipid metabolism. Data are presented as means from four independent biological replicates. Column standardization was performed during data visualization. In this study, VIP values (VIP ≥ 1) combined with fold-change (FC) values (|log_2_FC| ≥ 1) were used to screen the differential accumulated metabolites. QR500, MIC quercetin-treated group; QR1000, 2 MIC quercetin-treated group; QR0, control group. LPE, lysophosphatidylethanolamine; LPC, lysophosphatidylcholine.

To further understand the global effects of quercetin treatment on *S. aureus* metabolites, we performed a liquid chromatography–mass spectrometry (LC–MS)-based metabolomic study. A total of 1131 metabolites were detected and were classified based on a qualitative analysis, as shown in Figure 4A. The three most abundant metabolites were amino acids and their metabolites (38.73%), organic acids and their derivatives (12.38%), and benzene and its derivatives (9.02%). Lipids, including fatty acids, glycolipids, glycerophospholipids, and sphingolipids, were the fourth most prominent and numerous metabolites identified, accounting for 8.22%.

The metabolomic profiles of the control and the two quercetin-treated groups were subjected to unsupervised (principal component analysis, PCA) and supervised (orthogonal partial least-squares-discriminant analysis, OPLS-DA) multivariate analysis to determine the possibility of distinguishing between the two. The PCA score plot demonstrated significant differences for the metabolites of *S. aureus* treated with quercetin, and these metabolites were grouped into three distinct clusters based on the concentration of quercetin used (Figure 4B). The score plots generated via OPLS-DA also revealed a clear separation between the control group and the groups treated with 1 and 2 MIC of quercetin (Figure 4C,D). These findings indicated that the treatments of quercetin caused pronounced alterations in the metabolite profile of *S. aureus*. Using criteria of variable importance in projection (VIP) values greater than 1 and fold-changes (FC) exceeding an absolute value of 1 (|log_2_FC| > 1), 489 differentially accumulated metabolites (DAMs) between the MIC quercetin treatment group and the control were identified. Of these, 287 DAMs exhibited increased abundance after the treatment, while 202 DAMs demonstrated reduced concentrations. Likewise, a comparison between the 2 MIC quercetin treatment and the control groups revealed 625 DAMs, with 371 being up-regulated and 254 being down-regulated (Figure 4E). Correlation analyses were performed on all the DAMs. More evident differences at the DAM level were noted between the 2 MIC quercetin treatment and the control groups, whereas the differences between the MIC group and the control groups were less significant, indicating that the impact of quercetin treatment on the metabolites of *S. aureus* is positively correlated with the treatment concentration (Figure 4F).

At the same time, the use of liquid chromatography–tandem mass spectrometry (LC–MS/MS) technology allowed for a detailed analysis of changes in the relative content of phospholipids. In total, ten lysophosphatidylethanolamines (LPEs) and eight lysophosphatidylcholines (LPCs) were detected (Figure 4G). All 10 LPEs were up-regulated following the 2 MIC quercetin treatments, with an average up-regulation of 43.16%. LPE (18:2/0:0) was the most up-regulated, with an increase of 72.98%. The top three most up-regulated LPEs all contained an unsaturated fatty acid chain. Compared to the control group, five of the eight LPCs were up-regulated following the 2 MIC quercetin treatments, with LPC (12:0/0:0) being the most up-regulated, with an increase of 85.61%. Evidently, *S. aureus* accumulated more LPCs and LPEs under the stress of quercetin.

### 2.3. Gene Expression Analysis of S. aureus in Response to Quercetin Treatments

#### 2.3.1. Transcriptome Analysis

**Figure 5:**
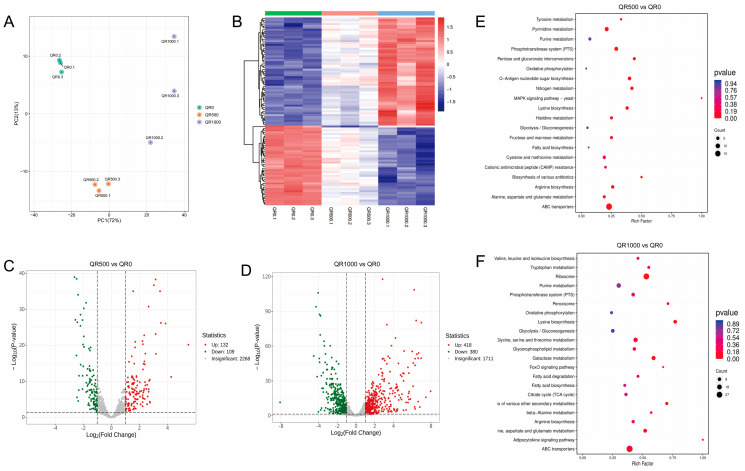
Transcriptome analysis of *S. aureus* ATCC 27217 under different quercetin treatments. (**A**) PCA score plot. (**B**) Clustering heatmap of differentially expressed genes (DEGs). (**C**,**D**) Volcano plots of DGEs. Each point in the graph represents a gene; green represents differentially down-regulated genes, red represents differentially up-regulated genes, and gray represents genes with insignificant differences. (**E**,**F**) Kyoto encyclopedia of genes and genomes (KEGG) pathway enrichment analysis of DEGs. DEGs were selected based on a VIP > 1 and |log_2_ (fold-change)| > 1.

A transcriptomic analysis was conducted to investigate the genetic mechanism of *S. aureus* in response to quercetin treatments. Transcriptome sequencing yielded a total of 134,792,318 clean reads (constituting 20.63 GB of clean data). The guanine–cytosine (GC) percentage was 35.71%, and the Q30 percentage (with a sequencing error rate < 0.1%) was 91.83%. This high sequencing quality met the requirements for subsequent analyses. PCA was used to analyze the differences in gene expression among the three groups (Figure 5A). The samples within each group showed high similarity, ensuring the reliability of the subsequent differential gene analysis.

The correlation heatmap (Figure 5B) shows that the expression of some differentially expressed genes (DEGs) decreased as the concentration of quercetin increased, while that of others increased, suggesting that quercetin’s impact on the gene expression of *S. aureus* is dose-dependent. In comparison to the control group, the group treated with the MIC of quercetin had 241 differentially expressed genes (|log_2_FC| > 1, false discovery rate, FDR < 0.05), with 132 up-regulated and 109 down-regulated DEGs (Figure 5C). Among the 798 DEGs between the 2 MIC group and the control group, 418 genes were up-regulated and 380 genes were down-regulated (Figure 5D). To identify the differentially expressed genes involved in metabolic pathways, Kyoto encyclopedia of genes and genomes (KEGG) enrichment analysis was applied to all the DEGs. As shown in Figure 5E,F, the number and significance of genes enriched in the fatty acid biosynthesis pathway escalated as the concentrations of quercetin levels increased.

#### 2.3.2. RT-qPCR Validation of the Transcriptomic Data

Based on the results of transcriptome sequencing and pathway enrichment analyses, a total of seven differentially expressed genes were selected for verification of the transcriptome sequencing data via RT-qPCR. These included five genes involved in fatty acid biosynthesis (*fabG*, *fabF*, *fabD*, *fabZ*, and *fadD*), one gene involved in ABC transporter activity (*malK*), and one gene involved in energy metabolism (*sdhC*). The RT-qPCR results indicated that the expression patterns of all the selected DEGs were consistent with the transcriptome analysis, suggesting that the transcriptome analysis was convincing (Figure S2).

### 2.4. Combined Analysis of the Metabolomic and Transcriptomic Data Related to Energy Metabolism and Fatty Acid Biosynthesis Pathways

Cellular-level evidence has shown that quercetin treatment affects intracellular energy and cell membrane function in *S. aureus*. The identification of genes and metabolites in the related pathways responsive to quercetin treatment is pivotal for comprehending the inherent mechanisms governing *S. aureus* responses.

**Figure 6:**
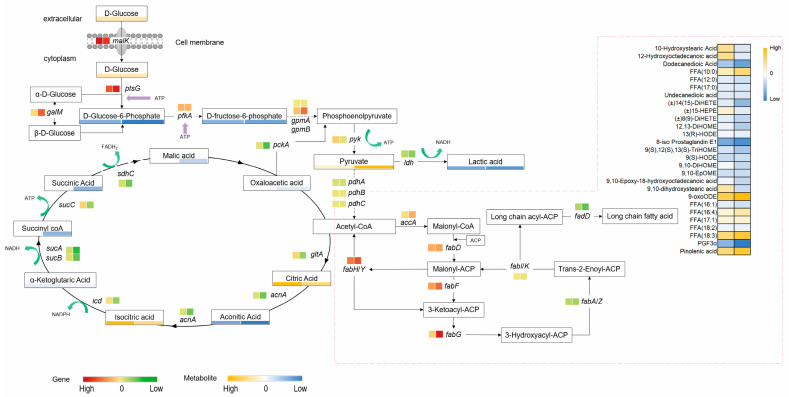
Involvement of DAMs and DEGs in the fatty acid metabolism and energy metabolism pathways of *S. aureus* ATCC 27217 under different quercetin treatments. Square box indicates DEGs; rectangular box indicates DAMs; left box, MIC quercetin-treated group vs. control group; right box, 2 MIC quercetin-treated group vs. control group. Data are presented as means from four independent biological replicates. Column standardization was performed during data visualization.

Based on the metabolome and transcriptome results, in the comparative analysis of the MIC, 2 MIC, and control groups, 15 and 49 differentially expressed genes, along with 17 and 20 differentially accumulated metabolites, respectively, were mapped to seven KEGG pathways related to energy metabolism. Four genes (*ptsG*, *galM*, and *gpmA/B)* involved in the Embden–Meyerhof–Parnas (EMP) pathway (glycolysis) were found to exhibit various degrees of overexpression in the quercetin-treated groups (Figure 6). For instance, the expression of *ptsG*, which codes for the glucose phosphotransferase system (PTS) system protein, increased 2.67-fold, suggesting a positive response of glycolysis in *S. aureus* treated with quercetin. The expression levels of key genes, such as *gltA*, *acnA*, *icd*, *sucA*, *sucB*, *sucC*, and *sdhC*, were significantly down-regulated in the tricarboxylic acid cycle (TCA) cycle following treatment with 2 MIC of quercetin. Furthermore, the relative levels of several critical intermediates, including aconitic acid, succinic acid, malic acid, and oxaloacetic acid, were significantly reduced. These results indicate that quercetin inhibited the TCA cycle in *S. aureus*.

Lipid metabolism encompasses the biochemical processes involved in the synthesis and degradation of lipids in the cell [34]. Fatty acid metabolism, an important subset of lipid metabolism, refers specifically to the processes of fatty acid biosynthesis and oxidation. Five and twenty-one DEGs were identified from the comparisons of the MIC, 2 MIC, and control groups, respectively. These DEGs were then mapped to the relevant pathways of lipid metabolism. In the fatty acid biosynthesis pathway of *S. aureus* treated with 2 MIC of quercetin, the expression of *accA*, *fabD*, *fabF*, and *fabH* was up-regulated, while the expression of *fabA/fabZ*, *fabI/K*, and *fadD* was down-regulated to various extents. As a turning point for changes in gene expression, it was suggested that *fabG* might be a target of quercetin action.

**Figure 7:**
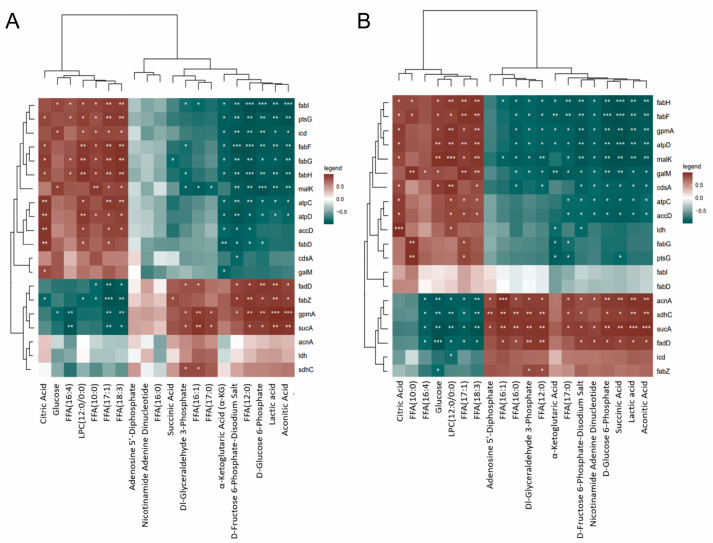
Spearman correlation heatmap of DAMs and DEGs associated with the fatty acid metabolism and the energy metabolism pathways of *S. aureus* ATCC 27217 under different quercetin treatments. (**A**) MIC quercetin-treated group vs. control group; (**B**) 2 MIC quercetin-treated group vs. control group. The Spearman correlation coefficient is represented by the color of each cell in the heatmap, with red represents a positive correlation and green represents a negative correlation. The intensity of the color denotes the strength of the correlation. A significant correlation is confirmed if the *p*-value with the Bonferroni correction is less than 0.05 (*), 0.01 (**), or 0.001 (***).

However, the metabolomic data could not be directly mapped to the fatty acid biosynthesis pathway because the intermediates of this pathway were all connected with the acyl-carrier-protein. To accurately elucidate the effects of quercetin on the energy and lipid metabolism of *S. aureus*, the corresponding mRNA and metabolites were normalized and subjected to statistical analysis. The corresponding correlation cluster heatmap (Figure 7) shows the correlations between the expressed genes and metabolites of *S. aureus* in the control group and the quercetin-treated groups. The color intensity of each cell in the heatmap represents the Spearman correlation coefficient. We found a strong correlation between the transcriptomic and metabolomic data for energy metabolism but only a weak correlation for lipid metabolism. It is proposed that, in addition to the fatty acid biosynthesis pathway, quercetin may exert an effect on other pathways, subsequently affecting the bacterial fatty acid composition. Furthermore, it is hypothesized that quercetin influences the bacterial fatty acid composition not only at the transcriptional level but also potentially at the protein level.

### 2.5. Inhibition of β-Ketoacyl-ACP Reductase by Quercetin

#### 2.5.1. Quercetin’s Inhibitory Potency and Type of Inhibition toward the FabG Enzyme

The joint analysis of the metabolomic and transcriptomic data suggests that *fabG* and thus the enzyme encoded by it may be a potential target used by quercetin against *S. aureus*. Quercetin’s inhibitory effect on FabG was measured in vitro. The results of the enzyme activity assay demonstrated that quercetin inhibited FabG in a concentration-dependent manner, with an IC_50_ value of 76.36 μM, while the positive control, resveratrol, showed an IC_50_ value of 51.12 μM (Figure S3).

**Figure 8:**
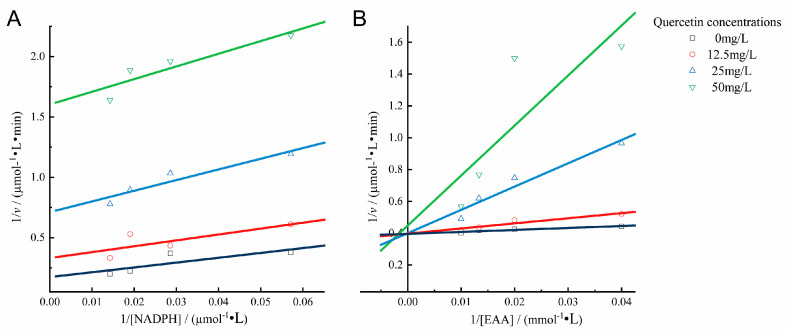
Lineweaver–Burk plots for the inhibition of FabG by quercetin. (**A**) The ethyl acetoacetate (EAA) concentration was fixed at 200 mmol/L, while the nicotinamide adenine dinucleotide phosphate (NADPH) was the variable substrate; its concentrations were tested at 17.5, 35, 52.5, and 70 μmol/L. (**B**) The NADPH concentrations was fixed at 35 μmol/L, while the EAA was the variable substrate; its concentrations were tested at 25, 50, 75, and 100 mmol/L. The concentrations of the inhibitor (quercetin) were 0, 12.5, 25, and 50 mg/mL. Data are presented as means from three repeated experiments.

Lineweaver–Burk plots were used to determine the types of inhibition shown by quercetin against FabG. The regression lines obtained at different concentrations of quercetin with respect to nicotinamide adenine dinucleotide phosphate (NADPH) appear as a set of parallel lines, with both *K*_m_ and *V*_max_ decreasing proportionally, illustrating a characteristic pattern of uncompetitive inhibition by quercetin with respect to NADPH (Figure 8A). Figure 8B shows that the regression lines are linearly fitted and intersect in the positive direction of the *y*-axis. The *V*_max_ remained constant, while the *K*_m_ values increased, indicating that quercetin acted as a competitive inhibitor with respect to the substrate ethyl acetoacetate (EAA).

#### 2.5.2. Quercetin’s Binding Mode with Respect to FabG via Molecular Docking Simulation

**Figure 9:**
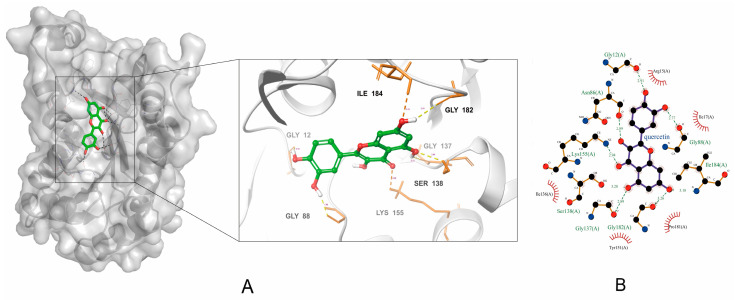
Molecular docking results of quercetin with the FabG–NADP^+^ complex in (**A**) three-dimensional and (**B**) two-dimensional diagrams. For clarity, only interacting residues are labeled. Dashed lines, hydrogen bonding interactions; half red circle, non-ligand residues involved in the hydrophobic contacts.

To further understand the inhibitory mechanism, the binding energy, binding sites, and the interaction forces of quercetin with respect to FabG were investigated through molecular docking simulations. The optimal docking results for quercetin with respect to the FabG–NADP^+^ complex is shown in Figure 9. Quercetin exhibited a binding score of −7.541 kcal/mol. We observed various binding interactions between the quercetin molecule and amino acid residues of FabG. Specifically, the C-5 hydroxyl group of the quercetin molecule formed two hydrogen bonds with Gly-137 and Ser-138, while the C-7 hydroxyl group established two hydrogen bonds with Gly-182 and Ile-184, and hydrogen bonds between the C-3 hydroxyl group and Asn86, C-4 carbonyl and Lys-155, C-4′ hydroxyl group and Gly-12, and C-5′ hydroxyl group and Gly-88 were also observed. In addition, the quercetin molecule engaged in hydrophobic interactions with Tyr-151, Pro-181, Ile-136, Ile-17, and Arg-15. These interactions ensure that the compound is firmly bound with the protein.

## 3. Discussion

Energy metabolism supplies the energy and raw materials necessary for bacteria to synthesize new cellular components, such as proteins, nucleic acids, and lipids. The results of the present study suggest that quercetin caused an energy deficit in *S. aureus*, as evidenced by the significant decrease in the ATP content. The results pertaining to the metabolome and transcriptome, annotated using KEGG, indicate that the quercetin treatments inhibited the TCA cycle of *S. aureus* and enhanced the glycolytic pathway. The total intracellular ATP content decreased significantly due to the lower ATP production efficiency of the glycolytic pathway compared to that of the TCA pathway. The synthesis of fatty acids is an energy-intensive process [35,36]; under energy-limited conditions, bacteria may reduce metabolic pathways that require large energy inputs, such as fatty acid biosynthesis. Acetyl-coenzyme A (acetyl-CoA) is essential for bacterial fatty acid biosynthesis, providing the two-carbon unit needed for chain elongation. Its primary source is pyruvate oxidation, linking energy metabolism to fatty acid biosynthesis. The pyruvate dehydrogenase complex catalyzes the decarboxylation of pyruvate to form acetyl-CoA [37]. In this study, the expression levels of the pyruvate-dehydrogenase-encoding genes *pdhA*/*B*/*C* did not exhibit significant differences under the treatments of quercetin, indicating that, in the case of an abnormal accumulation of pyruvate, the suppression of the TCA cycle reduced the metabolic flux into this pathway, lowering acetyl-CoA production. This is also evidenced by the down-regulation of the encoded gene of citrate synthase, playing a key role in initiating the TCA cycle by converting oxaloacetate and acetyl-CoA to citrate. Although acetyl-CoA can also be derived from β-oxidation, amino acid degradation, and alcoholic fermentation [38], the hindered energy pathways under quercetin’s influence restrict major acetyl-CoA availability, adversely impacting bacterial fatty acid biosynthesis. NADPH is critical for fatty acid biosynthesis, acting as a reducing agent. The pentose phosphate pathway (PPP) is a key source of NADPH, converting glucose-6-phosphate into ribose-5-phosphate and NADPH [39]. However, quercetin diverts glucose-6-phosphate to the glycolytic pathway, decreasing its availability in the PPP and subsequently reducing NADPH production, leading to insufficient NADPH available for participation in the fatty acid biosynthesis pathway.

In this study, we confirmed, through observations made using scanning electron microscopy and confocal laser scanning microscopy, combined with measurements of intracellular substance leakage, that the integrity of the cell membrane of *S. aureus* was compromised after treatment with quercetin. Metabolite analysis using GC–MS technology combined with a standard product comparison provided an accurate quantification of 14 fatty acids in quercetin-treated *S. aureus*. The results showed that under MIC quercetin treatment, the levels of most fatty acids increased, demonstrating that even under conditions of limited energy, two-carbon units, and reducing power, bacteria still manage to increase the synthesis of fatty acids. The UFA/SFA ratio remained stable under MIC treatment but increased significantly at 2 MIC, with a more pronounced reduction in SFA levels. Unsaturated fatty acids (UFAs) increase the fluidity of cell membranes; a high UFA/SFA ratio makes the bacterial membrane softer and more flexible and might increase the ability of certain small molecules and ions to penetrate the membrane [33]. LC–MS/MS analysis showed that under quercetin treatment, *S. aureus* exhibits increased levels of LPCs and LPEs, impacting membrane viscosity and functions. The rise in the levels of these metabolites suggests a looser lipid arrangement due to larger hydrophilic head groups in phosphatidylcholine and phosphatidylethanolamine, reducing membrane hydrophobicity, which would probably diminish the passive permeation of hydrophobic molecules [40]. However, the fluidity of the cell membrane increased accordingly. Transcriptome analysis showed that *S. aureus* responded to quercetin stress by up-regulating gene expression in the fatty acid biosynthesis pathway. This aligns with the findings presented in other studies. Wang et al. suggested that naringenin induces the up-regulation of *S. aureus* fatty-acid-biosynthesis-related genes [41]. A study by Xu et al. showed a positive correlation between *fabF* gene expression and the free fatty acid content via an analysis of C13-labeled glucose metabolic streams [42]. However, it is noteworthy that the Spearman correlation coefficient obtained from the correlation study of the expressed genes and metabolites related to lipid metabolism suggested that the effects of quercetin on bacterial fatty acid biosynthesis transcended gene expression. Pathway mapping showed that in quercetin-treated *S. aureus*, the genes with up-regulated expression were mainly located in *fabG* and its preceding catalytic steps. Conversely, its downstream gene expression decreased. This suggested that *fabG* and its coded FabG are potential targets of quercetin, marking a significant shift in gene expression and possible action sites regarding quercetin’s influence on bacterial fatty acid biosynthesis pathways. As mentioned in the introduction, FabG is a promising target for the development of antimicrobial agents. This study suggests that *S. aureus* may attempt to counteract the effects of quercetin by enhancing the fatty acid biosynthesis pathway. Although the expression levels of genes active in steps prior to the *fabG* step, as well as *fabG* itself, were up-regulated, the enzymatic catalytic function of FabG was inhibited by quercetin, preventing the production of sufficient β-hydroxyacyl-ACP for subsequent steps. This explains the down-regulation of the expression levels of downstream genes. This would be one of the possible mechanisms by which quercetin causes abnormalities in the type II fatty acid biosynthesis pathway of *S. aureus*.

The inhibition kinetics and molecular docking simulations were then conducted to explore quercetin’s mechanism of action further. The kinetic analysis showed that quercetin was an uncompetitive inhibitor of FabG with respect to NADPH, indicating that quercetin prevented the detachment of NADP^+^ through the formation of ternary complexes consisting of the “enzyme–NADP^+^–compound”; concurrently, quercetin was a competitive inhibitor of FabG with respect to the substrate EAA, indicating that it competes with the substrate for the reactive site of FabG, thus preventing the binding of the enzyme to the substrate and achieving inhibition. Molecular docking simulations detailed quercetin’s action with respect to FabG. Binding to NADPH, FabG shifts significantly, creating an active-site tunnel for substrate catalysis. The critical active triad (Ser-138, Tyr-151, and Lys-155) at the tunnel’s base changes to active forms [22]. Quercetin enters this tunnel, forming hydrogen bonds, notably at positions C-4 and C-5 with Lys-155 and Ser-138, respectively. Other hydroxyl groups in quercetin’s structure further secure it within the tunnel through diverse interactions, highlighting quercetin’s dual inhibition mode: deterring NADP^+^ detachment and competing with the substrate at essential catalytic sites. This result strongly agrees with the inhibition mechanisms identified via our kinetic analysis. Tasdemir et al. investigated the inhibitory effects of 38 flavonoids on FabG [43]. This study stressed the importance of hydroxyl groups in the A and B rings for flavonoid efficacy. Compounds with dual hydroxyl groups in one ring exhibited superior FabG inhibition. The absence of a carbonyl group at C-4 negated any inhibitory effects. These findings are consistent with the role of each substituent in FabG inhibition by quercetin, as suggested in the molecular docking simulation analysis presented in this study.

To sum up, the underlying mechanisms of the quercetin-induced alteration of fatty acids are multifaceted. Quercetin affected bacterial energy metabolism and limited the availability of the energy supply, two-carbon units, and reducing power for fatty acid production. *S. aureus* enhances the expression of certain genes in the fatty acid biosynthesis pathway. However, quercetin directly inhibits the key reductase FabG, resulting in disruption of the *S. aureus* fatty acid biosynthesis pathway. A significant increase in the ratio of unsaturated to saturated fatty acids and an abnormal accumulation of membrane phospholipids directly contributed to the aberrant function of the bacterial cell membrane. Alteration of the fatty acid composition is one of the antibacterial mechanisms of flavonoids. They appear to have a variety of modes of action on bacteria, and in many cases, multiple mechanisms have been suggested for a single flavonoid compound, which is critical for combating bacterial resistance.

From a nutritional perspective, quercetin has been shown to have a wide range of biomedical benefits, including anti-carcinogenic, anti-inflammatory, anti-cancer, and anti-obesity activities [44]. Recent years have seen growing interest in the interaction between the gut microbiota and flavonoids. The relationship between the gut microbiome and flavonoids can be characterized as bidirectional. Gut microbiota play a crucial role in the absorption of dietary flavonoids. Moreover, flavonoids not only promote the growth of probiotics but also inhibit the growth of certain pathogenic bacteria, thereby enhancing the overall balance of the gut microbiota [45,46]. Considering the conserved nature of the fatty acid biosynthesis pathway in bacteria, the metabolomic and transcriptomic data from this study offer insights into the mechanisms by which flavonoids affect the gut microbiota.

A number of polyphenol-rich plant extracts, including green tea extract, grape seed extract, and pomegranate extract, have already been granted “generally recognized as safe” (GRAS) status, and their potential antimicrobial applications in the food industry have been extensively reviewed [47,48]. The main active ingredients are flavonoids and other natural phenolic compounds. Quercetin is a common dietary flavonol found in various fruits, vegetables, and tea beverages. It has minimal impact on the taste of foods and poses fewer risks to human health and the environment. Its potential for applications in the control of foodborne pathogens is demonstrated by its effect on *S. aureus* fatty acids and the underlying mechanisms, given the central role of fatty acids in bacterial physiology and the marked differences between the bacterial and human fatty acid biosynthesis pathways. Despite its potential, the minimum inhibitory concentration of quercetin against *S. aureus* reported in this study and others (ranging from 256 to 512 mg/L) indicates a high dosage requirement [49,50,51]. Numerous studies have demonstrated the synergy of flavonoids with currently used antimicrobial agents [10,52,53,54]. Some studies have been conducted, which have identified the potential synergistic effects of quercetin. Upon the addition of quercetin at concentrations ranging from 1 to 256 μg/mL, a significant reduction in the MIC was observed for tetracycline, oxytetracycline, chlortetracycline, and doxycycline against *E. coli*, with fractional inhibitory concentration (FIC) indices spanning a range from 0.094 to 0.5 [55]. Moreover, the combination of quercetin with amoxicillin has demonstrated the ability to augment cell membrane permeability, diminish fatty acid levels, and inhibit β-lactamase activity, thus effectively combating infections stemming from amoxicillin-resistant *Staphylococcus epidermidis* [56]. Therefore, a more effective approach would be to introduce previously known antibacterial substance with synergistic effects with quercetin, which can prevent pathogenic bacteria from bypassing the FAS-II synthesis pathway and synthesizing cell membrane phospholipids directly from exogenous long-chain fatty acids to achieve “bypass escape” [57,58]. Consequently, quercetin can act as a potentiator, reducing the MIC needed to inhibit foodborne pathogens, thus offering a strategy for combatting resistance. Furthermore, it can improve the nutritional properties of food.

## 4. Materials and Methods

### 4.1. Chemicals and Reagents

Quercetin (≥95%), resveratrol (≥95%), NADPH (99%), dimethyl sulfoxide (DMSO, 99.8%), and all fatty acid standards used in the GC–MS analysis were purchased from Sigma (Sigma-Aldrich, St. Louis, MI, USA). The chromatographically pure methyl tert-butyl ether (MTBE), acetonitrile and, methanol (MeOH), and n-hexane were obtained from Merck (Merck, Darmstadt, Germany), and bacterial culture media and all other analytically pure reagents were purchased from Sinopharm (Sinopharm, Shanghai, China). Ultrapure water was obtained using a PALL Lab Water Purification System (Port Washington, NY, USA).

### 4.2. Bacterial Strain and Culture Condition

*Staphylococcus aureus* ATCC 27217 was purchased from the China Industrial Microbial Culture Preservation Center (Beijing, China). *S. aureus* was cultured in Luria–Bertani (LB) broth for 12 h at 37 °C with constant shaking at 180 rpm. Next, the bacterial pellets obtained via centrifugation (4000 rpm, 5 min, 4 °C) were washed with saline and resuspended in saline to 10^8^ CFU/mL for further study.

For the quercetin treatment, the compound was dissolved in DMSO and then further diluted in LB broth as a stock solution to achieve a concentration of 10 mg/mL. The solution was filtered using a 0.22 µm filter (Millipore, Burlington, MA, USA) before use. Quercetin solution was added to the culture treatment medium, and the final concentrations of DMSO did not exceed 0.5% (*v*/*v*). *S. aureus* cells were treated with quercetin at concentrations of 250, 500, and 1000 mg/L for 24 h at 37 °C. The bacterial cells and supernatants were collected via centrifugation (4000 rpm, 10 min, 4 °C) for subsequent assays.

### 4.3. Minimum Inhibitory Concentration Assay

The minimum inhibitory concentration of quercetin against *S. aureus* ATCC 27217 was determined using the microdilution method in 96-well plates according to the protocol described by the Clinical and Laboratory Standards Institute (CLSI) [59]. The concentrations tested ranged from 0.25 to 4 mg/mL. Untreated bacteria and bacteria treated with DMSO (0.5% *v/v*) were used as blank and negative controls, and kanamycin sulfate (0.25 mg/L) was used as a positive control. The lowest concentration without bacterial growth was determined as the MIC after incubation at 37 °C for 24 h.

### 4.4. Scanning Electron Microscopy and Confocal Laser Scanning Microscopy

The potential effects of quercetin on *S. aureus* cell membrane integrity and permeability were determined via scanning electron microscopy and confocal laser scanning microscopy [60]. For scanning electron microscopy, the collected cells were fixed with 2.5% glutaraldehyde overnight at 4 °C, washed three times with PBS, and dehydrated through a graded ethanol series (30%, 50%, 70%, 90%, and 100% *v/v*). Afterward, they were dried and gold-plated. A Gemini SEM 360 scanning electron microscope (Zeiss, Oberkochen, Germany) was used for image acquisition. For confocal laser scanning microscopy, the bacterial cells were stained using the AO/PI double stain kit (CC2260, G-CLONE, Beijing, China). The cells were suspended in 500 μL of buffer from the kit. Next, 10 μL of a 1:1 mixture of acridine orange and propidium iodide was used to stain the cells. The cells were then incubated in the dark at 4 °C for 15 min, washed three times with PBS, and observed using a STELLARIS 5 laser scanning confocal microscope (Leica, Wetzlar, Germany).

### 4.5. Bacterial Protein and Nucleic Acid Leakage

The assays for bacterial protein and nucleic acid leakage were conducted following the methods previously described [61,62], with modifications. The protein concentration was determined using the BCA protein concentration assay kit (Biorigin, Beijing, China) with a Victor X4 multilabel plate reader (PerkinElmer, Waltham, MA, USA) according to the manufacturer’s instructions. For the measurements of nucleic acid leakage, the optical density at 260 nm was measured with an UV-2100 spectrophotometer reader (PerkinElmer, Waltham, MA, USA).

### 4.6. Intracellular ATP and Pyruvate Contents

The intracellular ATP and pyruvate contents were measured using an ATP assay kit (Nanjing Jiancheng Bioengineering Institute, Nanjing, China) and pyruvate assay kit (Bioseth Biotechnology, Zhenjiang, China), respectively, according to the manufacturers’ protocol. The intracellular concentrations of ATP and pyruvate were normalized to the protein concentration of the samples. Protein concentrations were determined using the Bradford assay [63].

### 4.7. Determination of Fatty Acid Composition

The bacterial cell fatty acid composition was determined via GC–MS. The collected *S. aureus* cells were resuspended in 100 μL of ultrapure water; 50 μL of the cell suspension was mixed with 75 μL of a methanol solution, 100 μL of a methyl tert-butyl ether solution, and 25 μL of a 36% phosphoric acid solution. After vortexing for 3 min, the mixture was immersed in liquid nitrogen for 2 min; this process was repeated twice, and then, the sample was centrifuged at 12,000 rpm for 5 min at 4 °C. The samples were subjected to fatty acid methyl esterification using the boron trifluoride method [64]. The fatty acid methyl esters were extracted with hexane for GC–MS analysis.

The samples were analyzed via Agilent 7890B gas chromatography coupled to an Agilent 7000D triple quadrupole mass spectrometry operated in electron impact (EI) ionization mode (GC–EI–MS/MS system, Agilent, Santa Clara, CA, USA). The analytical conditions were as follows: GC: column, DB-5MS capillary column (30 m × 0.25 mm × 0.25 μm, Agilent, Santa Clara, CA, USA); carrier gas, high-purity helium (purity > 99.999%); the heating procedure was started at 40 °C (2 min) and was increased at 30 °C/min to 200 °C (1 min), was increased at 10 °C/min to 240 °C (1 min), and was increased at 5 °C/min to 285 °C (3 min); traffic: 1.0 mL/min; inlet temperature: 230 °C; injection volume: 1.0 μL. EI–MS/MS: temperature 230 °C; ionization voltage: 70 eV; transmission line temperature: 240 °C; four-stage rod temperature: 150 °C; solvent delay: 4 min; scanning mode: selected ion monitoring (SIM). The fatty acids detected were qualified and quantified according to the method of the external standard, and the contents were normalized to the protein concentration of the samples, which were determine by using the BCA Protein Assay Kit mentioned above.

### 4.8. Metabolomics Analysis

Intracellular metabolites of *S. aureus* were extracted, and analyses were carried out as documented in the previous studies [65,66]. In brief, 40 mg of the bacterial cell sample was mixed with 500 μL of an 80% methanol solution (*v/v*) and an internal standard, vortexed, and subjected to three freeze–thaw cycles. After centrifugation, 200 μL of the supernatant was analyzed using a LC–MS/MS system. The analytical conditions were as follows: UPLC, ExionLC AD (Sciex, Framingham, MA, USA); column, Waters ACQUITY UPLC HSS T3 C18 (1.8 µm, 2.1 mm × 100 mm) (Waters, Milford, MA, USA); column temperature, 40 °C; flow rate, 0.4 mL/min flow rate, 2 μL injection, with a water and acetonitrile gradient. Linear ion trap (LIT) and triple quadrupole scans were acquired on a QTRAP^®^6500 triple quadrupole-linear ion trap mass spectrometer system (Sciex, Framingham, MA, USA), equipped with an electrospray ionization (ESI) turbo ion-spray interface, operating in positive and negative ion mode and controlled by Analyst 1.6.3 software (Sciex, Framingham, MA, USA).

Based on the self-built metabolites database and public biochemical databases, such as the Metlin database (https://metlin.scripps.edu/) (accessed on 29 March 2024), qualitative analysis was performed according to the retention time (RT), ion-pair information, and secondary spectrum data. Quantitative analysis of metabolites was performed based on the multiple reaction monitoring mode (MRM) of triple quadrupole mass spectrometry. Principal component analysis and orthogonal partial least-squares-discriminant analysis were undertaken by using the statistics function prcomp within R v3.5.0 (www.r-project.org) (accessed on 29 March 2024) and R package MetaboAnalystR software v4.3.2 (https://www.metaboanalyst.ca) (accessed on 29 March 2024), respectively. Differentially accumulated metabolites were determined based on the VIP (VIP > 1), *p*-value (*p* < 0.05, Students t-test), and absolute log_2_(fold-change) (|Log_2_FC| > 1). Identified metabolites were annotated using the KEGG compound database (http://www.kegg.jp/kegg/compound/) (accessed on 29 March 2024), and annotated metabolites were then mapped to the KEGG pathway database (http://www.kegg.jp/kegg/pathway.html) (accessed on 29 March 2024).

### 4.9. Transcriptomic Analysis

The total RNA of *S. aureus* cells was extracted using the RNAprep Pure Plant Kit (Tiangen, Beijing, China) according to the instructions provided by the manufacturer. RNA quantity was measured using a Qubit3.0 (Thermo Fisher Scientific, Waltham, MA, USA) and Nanodrop One (Thermo Fisher Scientific, MA, USA) at the same time. RNA integrity was accurately detected using the Agilent 4200 system (Agilent Technologies, Waldron, Germany). Whole mRNAseq libraries were generated by Biomarker Technologies (Beijing, China) using the NEB Next^®^ Ultra™ Directional RNA Library Prep Kit for Illumina^®^ (New England Biolabs, Ipswich, MA, USA), following the manufacturer’s recommendations. The library was sequenced on the Illumina NovaSeq 6000 platform (lllumina, San Diego, CA, USA).

Fastp (v.0.23.2) (https://github.com/OpenGene/fastp) (accessed on 29 March 2024) was used to process the raw fastq format data to obtain clean reads. These reads were then mapped to the NCBI Rfam database (http://www.sanger.ac.uk/Software/Rfm/) (accessed on 29 March 2024), and rRNA sequences were removed using Bowtie2 (v2.33) (https://github.com/BenLangmead/bowtie2) (accessed on 29 March 2024). The clean and high-quality reads were subsequently aligned to the reference genome with Bowtie2 (v2.4.5) (http://bowtie-bio.sourceforge.net/bowtie2/index.shtml) (accessed on 29 March 2024). PCA and expression heatmaps were utilized to analyze the relationships between samples. DESeq2 (v1.34.0) (http://www.bioconductor.org/packages/release/bioc/html/DESeq2.html) (accessed on 29 March 2024) was used to identify differentially expressed genes between two groups, applying a threshold of FDR ≤ 0.05 and |log_2_FC| ≥ 1. Finally, KEGG analysis was performed to identify pathways in which the differentially expressed genes were enriched (http://www.kegg.jp/kegg/pathway.html) (accessed on 29 March 2024).

### 4.10. RT-qPCR Validation

Seven DEGs, *fabG*, *fabF*, *fabD*, *fabZ*, *fadD*, *malK*, and *sdhC* as representative genes of the fatty acid biosynthesis, ABC transporter, and energy metabolism pathways, were selected for the quantitative determination of their in vivo expression in *S. aureus* cells. The experimental setup for microbial strain culture and quercetin treatments was consistent with that for transcriptomic sequencing. RT-qPCR was performed following the instructions of the YBR Premix Ex Taq TM II reagent kit (LABLEAD, Beijing, China) on a LightCycler 480 PCR platform (Roche Applied Science, Switzerland), and data were analyzed using the 2^−ΔΔCt^ method [67]. All qPCR primers are listed in Table S1.

### 4.11. In Vitro FabG Enzyme Activity Assay

The FabG protein was prepared, extracted, and purified following the procedures described in the literature [68,69,70], with the assistance of Tianyi Huiyuan Life Science & Technology Inc, Beijing, China.

The in vitro FabG enzyme activity assay was carried out with slight modifications to the method described by Li et al. [69]. The β-ketoacyl reduction was determined by measuring the decrease in NADPH absorbance at 340 nm using the spectrophotometer reader mentioned above. The reaction mixture, which included 50 mM phosphate buffer (pH 7.0), 200 mM EAA, and 37.5 μM NADPH in a total volume of 2 mL, was pre-incubated at 37 °C for 5 min with the presence of quercetin at a concentration range of 6.25–50 mg/L. The reaction, which had the quercetin absent, served as a blank control, and resveratrol was used as a positive control. The reactions were initiated through the addition of 10 μL of FabG and incubated for 2 min at room temperature. The initial rate of the absorbance decrease at 340 nm was recorded and used to calculate the enzyme activity. The enzyme activity measured for the quercetin-treated groups was labeled as *A*_F_, while the enzyme activity measured in the blank control group was marked as *A*_0_. The inhibition rate was calculated using the following equation: (1 − *A*_F_/*A*_0_) × 100%.

The type of inhibition was determined with respect to the substrate EAA and the co-factor NADPH using a Lineweaver–Burk plot [71]. To investigate the inhibition mechanism with respect to EAA, FabG was incubated with a fixed and saturating concentration of NADPH (35 μmol/L) and different concentrations of quercetin (0, 12.5, 25, 50 mg/L) and EAA (25, 50, 75, 100 mmol/L). The inhibition mechanism with respect to NADPH was determined in a similar way, with a fixed concentration of EAA (200 mmol/L) and varying concentrations of quercetin (0, 12.5, 25, 50 mg/L) and NADPH (17.5, 35, 52.5, 70 μmol/L). Other reaction conditions were the same as those described earlier.

### 4.12. Molecular Docking

The protein structure of FabG (PDB ID: 1Q7B) was downloaded from the Protein Data Bank (http://www.rcsb.org/) (accessed on 29 March 2024). Only one subunit from the homotetrameric structure was kept for further analysis using PyMOL (https://pymol.org/) (accessed on 29 March 2024) [72]. Hydrogen atoms were added to the protein structure using the “h_add” command in PyMOL, and water molecules and the NADP^+^ co-factor were removed. The molecular structure of quercetin (CID: 5280343) was obtained from the PubChem database (https://pubchem.ncbi.nlm.nih.gov/) (accessed on 29 March 2024) and subjected to energy minimization using the MM2 force field in Chem3D 20.0 (Cambridge Soft, Cambridge, MA, USA), with 1000 iterations and an energy convergence criterion of 0.001 kcal/mol. Both the receptor and ligand files were converted to PDBQT format using AutoDockTools 1.5.7 (http://autodock.scripps.edu/) (accessed on 29 March 2024). A grid box with dimensions of 22.5 Å × 22.5 Å × 22.5 Å was centered at *x* = −11.96, *y* = 12.35, *z* = 8.41 to encompass the potential binding site. Molecular docking simulations were performed using AutoDock Vina 1.5.7 [73]. The docking results were visualized and analyzed using PyMOL and the LigPlot^+^ V2.2 (https://www.ebi.ac.uk/thornton-srv/software/LigPlus/) (accessed on 29 March 2024), focusing on protein–ligand interactions and residues within the binding pocket.

### 4.13. Statistical Analysis

For GC–MS, LC–MS, and RNA-Seq analysis, four biological replicates were performed for each group. All other experiments were conducted with three biological and three technical replicates for each treatment, unless stated otherwise. The results were expressed as the mean ± standard deviation (SD). The data were statistically analyzed using Origin 2024 software (Origin Lab Inc., Northampton, MA, USA). The statistical significance of the data was then tested via one-way analysis of variance (ANOVA) followed by Tukey’s multiple comparison test. Statistical significance was determined with a *p*-value threshold of less than 0.05. Correlation analysis was conducted using Pearson’s correlation test.

## 5. Conclusions

The underlying mechanisms of quercetin-induced fatty acid alterations in *Staphylococcus aureus* ATCC 27217 were investigated using metabolomic and transcriptomic approaches. The increased ratio of unsaturated to saturated fatty acids and the abnormal accumulation of membrane phospholipids were directly responsible for the disruption of the bacterial cell membranes. Quercetin inhibited the energy metabolism of *S. aureus*, resulting in the inability of the bacterial fatty acid biosynthesis pathway to obtain sufficient energy, two-carbon units, and reducing agents. The expression of genes involved in the bacterial fatty acid biosynthesis pathway of *S. aureus* was found to be differentially regulated under the treatments of quercetin, and, as a pivotal point, *fabG* indicated that its encoded protein, FabG, was a potential target for the action of quercetin in interfering with the bacterial fatty acid biosynthesis pathway of *S. aureus*. These results demonstrate a complex regulatory mechanism for the alteration of the fatty acid composition of *S. aureus* in response to quercetin, and they offer a molecular foundation for future research into the dual role of quercetin, a dietary flavonoid nutraceutical, in food applications as an antibacterial agent and a nutrition enhancer.

## Figures and Tables

**Table 1:** Fatty acid contents in quercetin-treated *S. aureus* ATCC 27217 cells.

Type	Fatty Acids	Contents under Different Quercetin Treatments (µg/mg prot)		
		0	MIC	2 MIC
SFA	Hexanoic acid	34.11 ± 0.49	42.57 ± 1.39	18.33 ± 2.98
	Octanoic acid	57.97 ± 2.00	69.73 ± 1.82	46.82 ± 9.24
	Nonanoic acid	68.96 ± 4.81	72.23 ± 3.79	35.78 ± 11.23
	Hendecanoic acid	18.43 ± 0.10	23.78 ± 0.61	9.10 ± 2.30
	Decanoic acid	117.91 ± 1.26	132.41 ± 7.84	114.90 ± 6.86
	Lauric acid	136.50 ± 1.16	126.07 ± 1.88	96.35 ± 5.50
	Tridecanoic acid	66.61 ± 2.24	47.70 ± 4.43	16.93 ± 5.15
	Myristic acid	2283.10 ± 58.53	1327.22 ± 188.88	831.96 ± 135.76
	Pentadecanoic acid	153.67 ± 1.19	128.14 ± 8.06	57.71 ± 34.86
	Palmitic acid	33,206.34 ± 246.36	38,876.56 ± 708.15	17,603.76 ± 563.07
	Heptadecanoic acid	217.40 ± 1.20	231.80 ± 3.18	142.77 ± 41.14
	Stearic acid	24,172.08 ± 97.55	29,193.94 ± 576.61	12,103.67 ± 222.55
UFA	Oleic acid	69,749.59 ± 1514.69	78,787.67 ± 1645.64	54,176.61 ± 1909.60
	Linoleic acid	332.25 ± 26.11	365.29 ± 25.03	351.40 ± 0.08
	Total SFA	60,533.10 ± 416.88	70,272.16 ± 1506.63	31,078.09 ± 1040.64
	Total UFA	70,081.83 ± 1540.80	79,152.96 ± 1670.67	54,528.01 ± 1909.68
	UFA/SFA ratio	1.16	1.13	1.75

SFA, saturated fatty acid; UFA, unsaturated fatty acid; prot, protein content determined by using the BCA protein concentration assay kit; MIC, the minimum inhibitory concentration of quercetin against *S. aureus* ATCC 27217, which was found to be 0.5 mg/mL. Values represent means ± SD (*n* = 4).

## Data Availability

The sequence data have been uploaded in the SRA database of National Center for Biotechnology Information (https://www.ncbi.nlm.nih.gov/sra/) (accessed on 5 April 2024) under the accession number of PRJNA1094409.

## Supplementary Materials

The following supporting information can be downloaded at: https://www.mdpi.com/article/10.3390/molecules29102266/s1, Figure S1: The molecular structure of quercetin, Figure S2: RT-qPCR validation of selected differentially expressed genes of *S. aureus* ATCC 27217 under different quercetin treatments, Figure S3: The inhibition rate of quercetin and resveratrol on FabG. Table S1: Primer sequences used for RT-qPCR analysis.
