# Supplementary material for: Integrated Metabolomic and Transcriptomic Analysis Reveals the Underlying Antibacterial Mechanisms of the Phytonutrient Quercetin-Induced Fatty Acids Alteration in Staphylococcus aureus ATCC 27217
Source: Molecules. 2024 May 11;29(10):2266. doi: 10.3390/molecules29102266 (PMC11123838; doi:10.3390/molecules29102266)
Supplement: Supplementary file 1 [file molecules-29-02266-s001.zip › molecules-2993025-supplementary.pdf]

Supplementary Materials

Contents

Figure S1 The molecular structure of quercetin.

Figure S2 RT-qPCR validation of selected differential expressed genes of *S. aureus* ATCC 27217 under different quercetin treatments.

Figure S3. The inhibition rate of quercetin and resveratrol on FabG.

Table S1. Primer sequences used for RT-qPCR analysis.

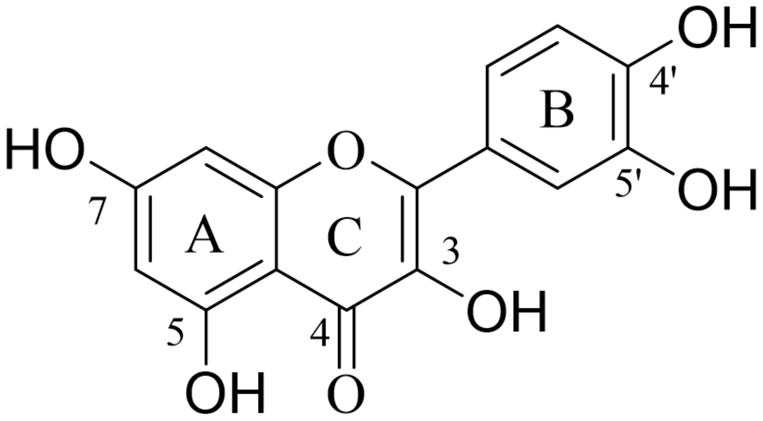

Figure S1 The molecular structure of quercetin.

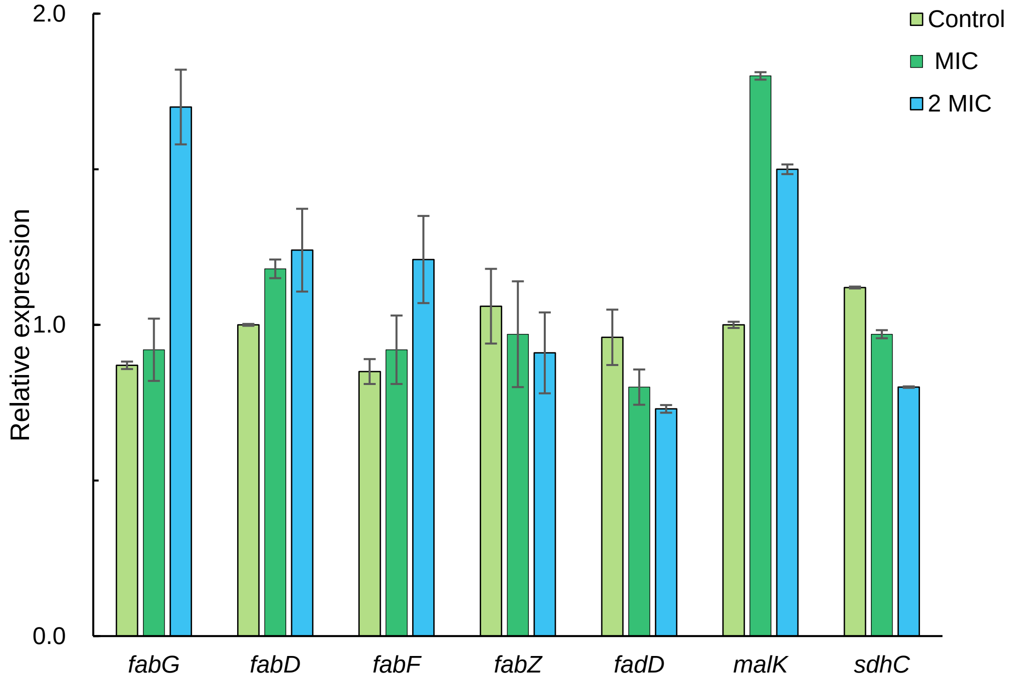

Figure S2 RT-qPCR validation of selected differential expressed genes of *S. aureus* ATCC 27217 under different quercetin treatments. Error bars represent the standard deviations (SD) from four repeated experiments.

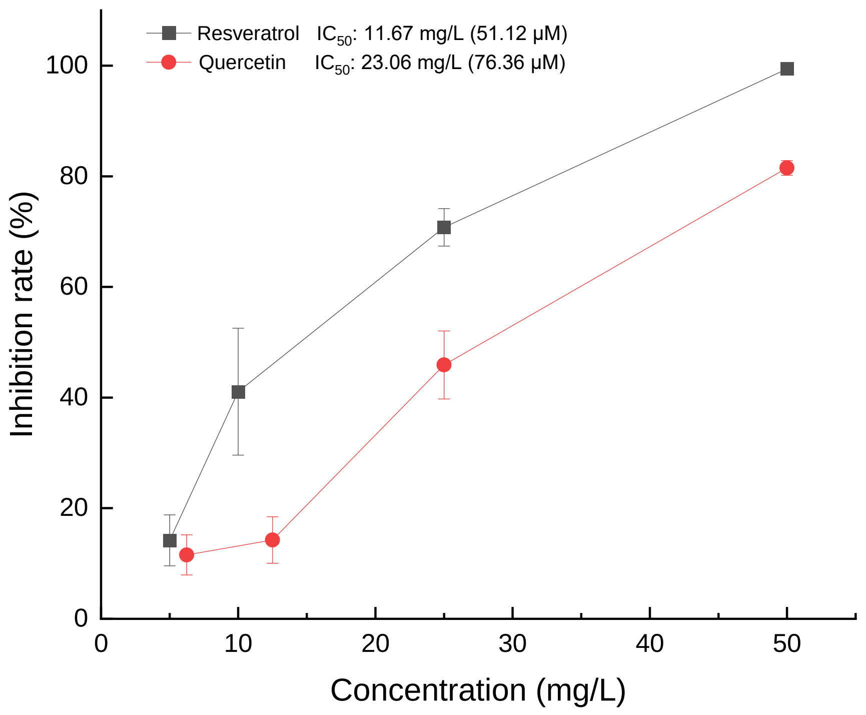

Figure S3. The inhibition rate of quercetin and resveratrol on FabG.

Table S1. Primer sequences used for RT-qPCR analysis.

Gene	Primer	Sequence（5′-3′）	Length
fabG	*fabG*-F	GTTGCCGATGCTGATGAAGT	137 bp
	fabG-R	TCATCCCACTCTTGTTCTTTCA	
fabD	*fabD*-F	TTGACGCATAGTTCGGCATT	197 bp
	fabD-R	ACTGCAGCCATGCTTCCTACA	
fabF	*fabF*-F	TTCTGGTATCGGTGGTATGGA	122 bp
	fabF-R	CTTGCCCAGTTGCCATATCA	
fabZ	*fabZ*-F	GGTATGGGCGTCGTTACAAGTA	145 bp
	fabZ-R	GCTATTAAAACGGGGATGATTG	
fadD	*fadD*-F	AGCAGAGACTGCCAAAGCAT	183 bp
	fadD-R	CATAGCCAACGACCACACAC	
malK	*malK*-F	TTGCTTTGGGCAGAGCTATT	160 bp
	malK-R	TGTGCCGACTTGCATAATGT	
sdhC	*sdhC*-F	CCTGTTGGATGCGCTATATGTC	181 bp
	sdhC-R	TCCAGCGAGCCATCAGGTAA	
16sRNA	16sRNA-F	CGGTGAATACGTTCYCGG	124 bp
	16sRNA-R	GGWTACCTTGTTACGACTT	
